# Supplementary material for: Functional characterization of a human epilepsy-associated gene network reveals metabolic regulation as a critical factor underlying seizure susceptibilities
Source: Dis Model Mech. 2026 Jan 28;19(1):dmm052307. doi: 10.1242/dmm.052307 (PMC12869506; doi:10.1242/dmm.052307)
Supplement: Supplementary information [file dmm-19-052307-s1.pdf]

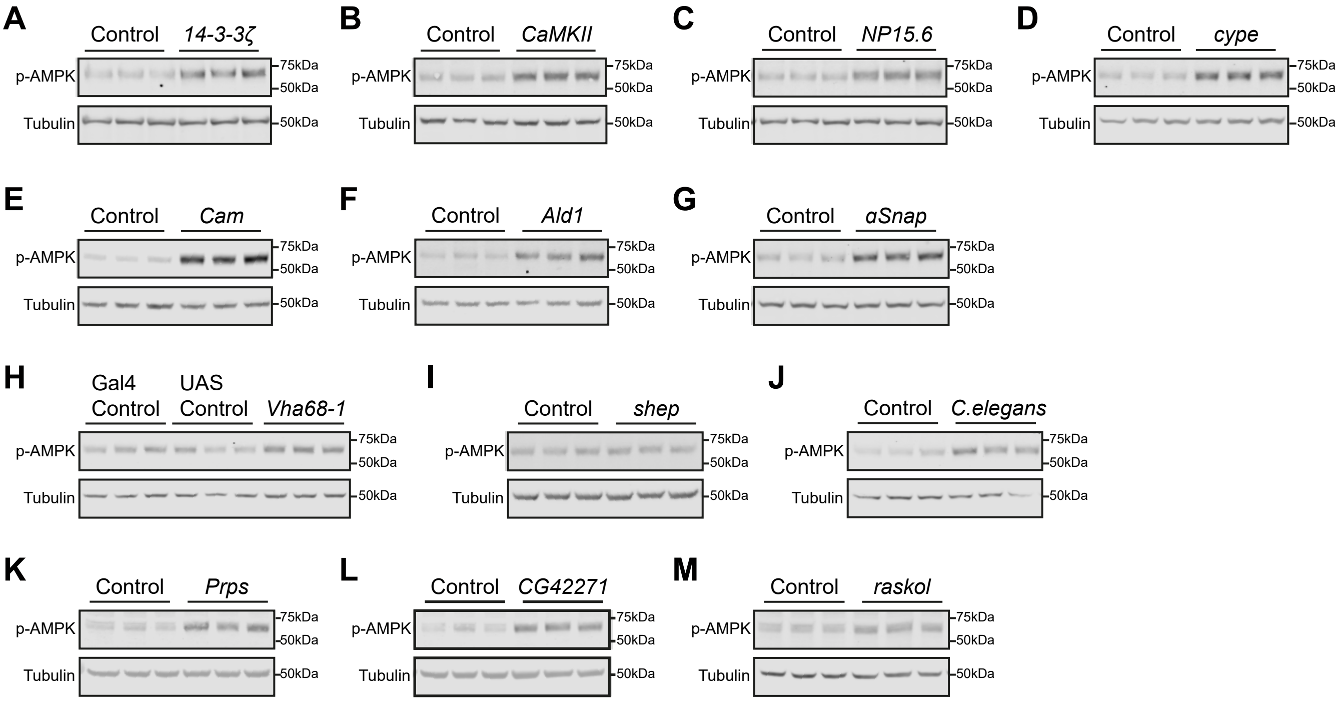


**Fig. S1. Additional western blots showing variable increases in AMPK phosphorylation in pan-neuronal knockdown models.** (A-M) Western blots showing three biological replicates of head homogenates from adult flies containing *nSyb-Gal4* and UAS-RNAi genetic elements or their genetic background controls using anti-phospho-AMPK (p-AMPK) and anti-tubulin antibodies. Quantification is shown in Fig. 5.

Disease Models & Mechanisms • Supplementary information

## Table S1. The *Drosophila* orthologs of 320 human genes in M30 cluster

Available for download at
https://journals.biologists.com/dmm/article-lookup/doi/10.1242/dmm.052307#supplementary-data

## Table S2. 287 selected *Drosophila* orthologs of M30 cluster

Available for download at
https://journals.biologists.com/dmm/article-lookup/doi/10.1242/dmm.052307#supplementary-data

## Table S3. 142 *Drosophila* genes for clustering analysis

Available for download at
https://journals.biologists.com/dmm/article-lookup/doi/10.1242/dmm.052307#supplementary-data

## Table S4. Hierarchically clustered coexpression matrix of 142 x 142 genes with pairwise Spearman correlation values

Available for download at
https://journals.biologists.com/dmm/article-lookup/doi/10.1242/dmm.052307#supplementary-data

## Table S5. Complete KEGG pathway and GO biological process terms list

Available for download at
https://journals.biologists.com/dmm/article-lookup/doi/10.1242/dmm.052307#supplementary-data

**Table S6. Epilepsy-associated gene list of M30 network**

Available for download at
https://journals.biologists.com/dmm/article-lookup/doi/10.1242/dmm.052307#supplementary-data

**Table S7. Crosses and genotypes in co-expression gene modules and associated lethality in pan-neuronal**

Available for download at
https://journals.biologists.com/dmm/article-lookup/doi/10.1242/dmm.052307#supplementary-data

**Table S8. Novel versus established links between genes of the evolutionarily conserved and co-expressed 26-gene module with altered seizure susceptibility, energy metabolism and AMPK activity. Abbreviations: AMPK – Adenosine monophosphate-activated protein kinase.**

Available for download at
https://journals.biologists.com/dmm/article-lookup/doi/10.1242/dmm.052307#supplementary-data
